# Mechanisms of Endoplasmic Reticulum Protein Homeostasis in Plants

**DOI:** 10.3390/ijms242417599

**Published:** 2023-12-18

**Authors:** Zhihao Duan, Kai Chen, Tao Yang, Ronghui You, Binzhao Chen, Jianming Li, Linchuan Liu

**Affiliations:** 1 State Key Laboratory for Conservation and Utilization of Subtropical Agro-Bioresources, Guangdong Key Laboratory for Innovative Development and Utilization of Forest Plant Germplasm, College of Forestry and Landscape Architecture, South China Agricultural University, Guangzhou 510642, China; 2 Department of Biology, Hong Kong Baptist University, Kowloon, Hong Kong

**Keywords:** ER homeostasis, unfolded protein response (UPR), ER-associated degradation (ERAD), ER-phagy, *Arabidopsis thaliana*

## Abstract

Maintenance of proteome integrity is essential for cell function and survival in changing cellular and environmental conditions. The endoplasmic reticulum (ER) is the major site for the synthesis of secretory and membrane proteins. However, the accumulation of unfolded or misfolded proteins can perturb ER protein homeostasis, leading to ER stress and compromising cellular function. Eukaryotic organisms have evolved sophisticated and conserved protein quality control systems to ensure protein folding fidelity via the unfolded protein response (UPR) and to eliminate potentially harmful proteins via ER-associated degradation (ERAD) and ER-phagy. In this review, we summarize recent advances in our understanding of the mechanisms of ER protein homeostasis in plants and discuss the crosstalk between different quality control systems. Finally, we will address unanswered questions in this field.

## 1. Introduction

**Figure 1:**
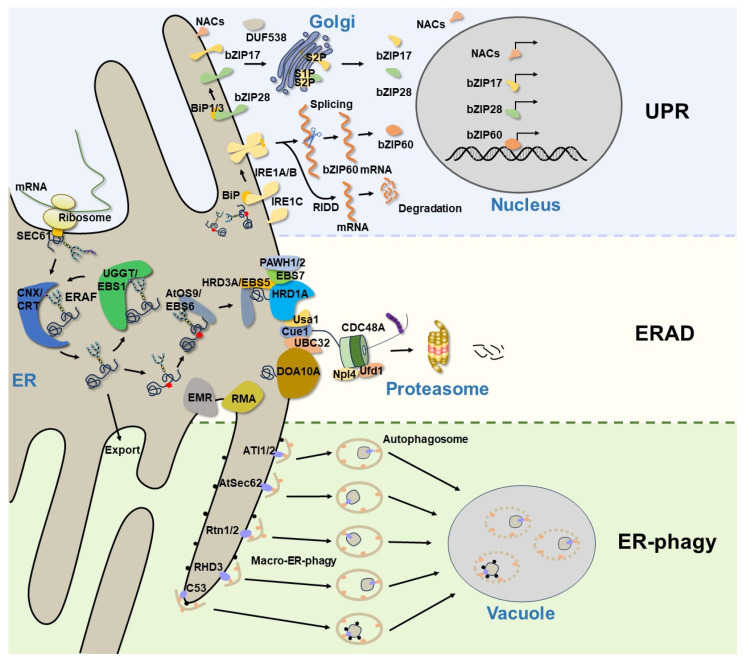
A current model of ER protein homeostasis in plants. The newly synthesized secretory and membrane proteins in the ER undergo folding and assembly, while those misfolded/unfolded proteins are handled by the ERAF mechanisms. The accumulation of misfolded proteins in the ER will trigger the UPR to relieve ER stress and restore ER homeostasis. In Arabidopsis, the UPR is modulated via the UPR sensor IRE1A/B, as well as via bZIP transcription factors (bZIP17, bZIP28, and bZIP60) and plant-specific NAC transcription factors. Terminally misfolded proteins are degraded by the ERAD system, which consists of many membrane-anchored E3 ligases and other conserved components. However, large protein aggregates or damaged ER segments that cannot be degraded by ERAD are eventually removed through ER-phagy. The ER-phagy receptors selectively recognize their cargoes and recruit them to the autophagosomes for autophagic degradation.

The endoplasmic reticulum (ER) is the largest intracellular organelle and plays essential roles in protein folding, lipid biosynthesis, detoxification, calcium storage, and carbohydrate metabolism [1,2]. In eukaryotic cells, nearly one-third of all proteins enter the secretory pathway via the ER. Only those proteins that are properly folded are allowed to leave the ER and be delivered to their final destinations. However, protein folding is a highly error-prone process that can be easily perturbed by a wide range of cellular and environmental stresses, leading to the accumulation of misfolded proteins and their aggregates in the ER, causing cellular dysfunctions or even cell death. To cope with this situation, eukaryotes have evolved many ER protein quality control (ERQC) systems to preserve ER proteostasis and to maintain cell survival [3]. In plants, ER stress usually occurs when they are subjected to unfavorable environmental conditions or at specific developmental stages. An evolutionarily conserved signal network, known as the unfolded protein response (UPR), is activated during ER stress to restore ER homeostasis [4]. In addition to transducing the ER stress signal to the nucleus to stimulate the expression of ER chaperones for ER-assisted protein folding/refolding (ERAF), the UPR also boosts the cellular capacity to degrade misfolded ER proteins through proteasomal and/or autophagic degradation [5,6]. ER-associated protein degradation (ERAD) is a well-characterized ER protein quality control mechanism that targets misfolded, improperly assembled, and even unwanted “correctly-folded” proteins for cytosolic proteasomal degradation. Genetic and biochemical studies on yeast and mammalian cells revealed sophisticated ERAD mechanisms and identified many ERAD components that are conserved from yeast to humans [7]. Recent genetic studies using the model plant *Arabidopsis thaliana* have also discovered a highly conserved ERAD complex [8,9]. Arabidopsis mutants with defects in ERAD components often exhibit abnormal responses to biotic or abiotic stress [8], demonstrating the necessity of the ERAD function in maintaining ER protein homeostasis during plant–environment interactions. Additionally, multiple lines of evidence have revealed that some stress conditions not only induce the UPR, but also activate the autophagy pathway to ensure the timely clearance of ER portions containing misfolded/aggregated proteins or damaged ER [10]. This means that the UPR exquisitely cooperates with ERAD and ER-phagy to maintain ER homeostasis, ER morphology, and ER function (Figure 1). Here, we will focus on recent advances in understanding the regulatory mechanisms of ER protein homeostasis and review the recent findings on the UPR, ERAD, and ER-phagy, suggesting an integration of ER protein quality control mechanisms during plant growth and development.

## 2. Activation of the UPR to Restore ER Homeostasis

The ER is the entry site for the secretory pathway. Newly synthesized secretory and integral transmembrane proteins enter the ER through the translocation channel in an unfolded state [11]. Upon entry into the ER, the luminal molecular chaperones and folding enzymes facilitate protein folding and complex assembly to attain native conformations and properly assembled complexes [12], while incomplete or misfolded glycoproteins are handled by the ERAF system, which promotes protein refolding by the ER folding-sensor enzyme, UDP-glucose: glycoprotein glucosyltransferase (UGGT, known as EBS1 in Arabidopsis), and the lectin-like molecular chaperones, calnexin (CNX)/calreticulin (CRT) [13,14] (Figure 1). However, the ERAF is an error-prone process that often fails, especially when interfered with cellular and environment stresses, causing excessive accumulation of misfolded proteins and improperly assembled complexes in the ER. This leads to ER stress and activates an evolutionarily conserved UPR signaling cascade. The UPR was initially described in yeast, and it was later discovered in other eukaryotic organisms. In metazoans, three ER transmembrane proteins, inositol-requiring enzyme 1 (IRE1) [15,16], activating transcription factor 6 (ATF6) [17], and protein kinase R (PKR)-like ER kinase (PERK) [18], have been identified as UPR receptors that sense misfolded proteins in the ER and initiate distinctive signaling cascades to increase the ER folding capacity, reduce protein synthesis rates, and boost ERAD efficiency. While the UPR pathway is highly conserved across diverse eukaryotic organisms, only two UPR branches, which are mediated by homologs of IRE1 and ATF6, have been characterized in plants [19] (Table 1). The Arabidopsis genome encodes two IRE1 homologs: AtIRE1A and AtIRE1B [20,21]. Both of them have conserved functional modules consisting of an ER luminal N-terminal sensor domain, a single transmembrane domain, and the cytosolic catalytic domain possessing the kinase and endo-ribonucleotidase (RNase) activities [20]. During ER stress, AtIRE1A and AtIRE1B can homodimerize and autophosphorylate to catalyze the unconventional cytoplasmic splicing of *BASIC LEUCINE ZIPPER60* (*bZIP60*) mRNA, producing an active nuclear-localized bZIP60 transcription factor to regulate the expression of UPR target genes [22,23] (Figure 1). AtIRE1B is widely expressed in whole plants, while AtIRE1A is mainly expressed in embryos and seeds [20], and they display different responses to biotic and abiotic stress [24]. AtIRE1C is a recently discovered Brassicaceae-specific IRE1 isoform [25,26], which contains only a transmembrane domain and a cytosolic region with kinase and ribonucleotidase domains. Although AtIRE1C lacks the sensor domain that is essential in other IRE1 isoforms, it still participates in the physiological UPR, specifically during gametogenesis in Arabidopsis [26]. It remains to be investigated how AtIRE1C senses the disturbed ER protein homeostasis. In addition, IRE1 is able to cleave cellular mRNAs, leading to their degradation through a process known as regulated IRE-dependent decay (RIDD) [27,28] (Figure 1), which is considered an efficient way to reduce the influx of proteins into the ER. Evidence has demonstrated that Arabidopsis IRE1s participate in the degradation of a subset of mRNAs encoding the secretory pathway proteins [29]. The other branch of the UPR signaling network in plants is composed of bZIP membrane-associated transcription factors. In Arabidopsis, AtbZIP17 and AtbZIP28 are two functional homologs of the metazoan ATF6, featuring a N-terminal cytosol-facing bZIP domain, a single transmembrane segment, and a C-terminal lumen-facing UPR-sensing domain [30,31]. Upon ER stress, AtbZIP17 and AtbZIP28 are translocated from the ER to the Golgi. The Golgi membrane-localized site 1 protease (S1P) and site 2 protease (S2P) sequentially cleave the two AtbZIP proteins to release their N-terminal bZIP transcriptional domains that can translocate into the nucleus to upregulate the expression of many UPR genes encoding protein chaperones, folding catalysts, and components of the ERAD machinery and autophagy pathway [30,31] (Figure 1). Although it seems that bZIP60 and bZIP17/bZIP28 work on two independent, parallel pathways, they still coordinately regulate numerous overlapping genes to alleviate ER stress and to enhance plant stress tolerance [32,33]. In recent years, some plant-specific NAC [no apical meristem (NAM), Arabidopsis transcription activation factor (ATAF1/2) and cup-shaped cotyledon (CUC2)] type transcription factors and DUF538 family proteins have been reported to be involved in the plant UPR pathway [34,35,36,37,38], suggesting that plants have developed unique strategies to cope with ER stress in response to a wide variety of biotic and abiotic stresses.

Although much is known about the downstream nuclear events of the UPR pathway [61,62], the mechanism through which plant UPR receptors sense the accumulation of misfolded proteins in the ER remains unclear. In *Saccharomyces cerevisiae*, a groove-like area was observed in the 3D structure of the yeast Ire1p luminal domain. It is generally believed that un/misfolded proteins directly bind the groove site formed by Ire1p oligomerization, leading to the activation of its cytosolic RNase activity [63]. However, a subsequent structural biology study with the human IRE1α suggested that the groove in its luminal domain is too small to bind misfolded proteins [64], raising the question of whether there is a direct binding between misfolded proteins and IRE1α in vivo. In mammalian cells, the chaperone protein BiP (binding immunoglobulin protein), an ER-localized member of the HSP70 family, can interact with the luminal domains of IRE1α and PERK to prevent the dimerization or oligomerization needed for their activation, thus inhibiting the UPR. Such BiP-IRE1α/PERK interaction also prevents BiP from binding its cochaperones [65]. Upon ER stress, BiP binds to un/misfolded proteins, leading to the liberation of IRE1α and PERK. The two liberated UPR sensors can subsequently dimerize/oligomerize to activate their respective catalytic activities, thus activating the UPR signaling cascades [65,66,67]. In recent years, a variety of IRE1α-binding proteins were discovered in mammals, some of which are involved in the posttranslational modifications of IRE1α to regulate its activity or protein stability [6]. However, little is known about the interacting proteins and the regulatory mechanisms of plant IRE1s. It was known that the mammalian BiP binds to ATF6 and prevents the ER-Golgi trafficking of ATF6 and that the titration of BiP by un/misfolded proteins under ER stress conditions allows ATF6 translocation into the Golgi where ATF6 is cleaved by S1P and S2P [68]. Similarly, two Arabidopsis BiP homologs, BiP1 and BiP3, were found to interact with the C-terminal intrinsically disordered regions of bZIP28 to retain it on the ER membrane [69]. The competitive binding of the BIPs with un/misfolded proteins release bZIP28 that can be translocated to the Golgi for the S1P-/S2P-catalyzed cleavage. Thus, BiPs plays important roles in the ER protein homeostasis, not only in ERAF but also in the recognition of misfolded proteins and the regulation of the UPR pathways. In addition to BiPs, recent studies also discovered interactions of the UPR sensors with protein disulfide isomerases (PDIs) [70,71,72], but how such bindings regulate the UPR mechanism to restore ER protein homeostasis remains to be explored.

## 3. Removal of Misfolded Proteins through ERAD

ERAD is a highly conserved protein quality control system responsible for sending terminally misfolded proteins for cytosolic degradation, thereby maintaining the ER protein homeostasis. Extensive genetic and biochemical studies in yeast and mammals have revealed that an elaborate ERAD process involves at least four interdependent steps: substrate recognition, retrotranslocation across the ER membrane, ubiquitination at the cytosolic side, and degradation by 26S proteasomes [7]. One of the best-studied ERAD mechanisms deals with misfolded glycoproteins, because a majority of secretory or transmembrane proteins are modified by asparagine (N)-linked glycosylation. It was believed that this ERAD machinery recognizes a unique N-glycan structure on the terminally misfolded glycoproteins. If a misfolded glycoprotein stays in the ER for too long engaging repeatedly futile folding attempts, its N-linked glycans are trimmed by folding-sensitive ER-localized α-1,2-mannosidase, known as homologous to α-mannosidase 1 (Htm1) in yeast and ER-degradation enhancing α-mannosidase-like proteins (EDEMs) in mammals [73,74], to expose the α-1,6 mannose residue on N-glycans. This special N-glycan is recognized by a unique lectin chaperone, known as OS9 (osteosarcoma amplified 9) in mammals and Yos9 (yeast homolog of OS9) in yeast, while the hydrophobic amino acid residues exposed on the surface of misfolded glycoproteins are captured by Sel1L (suppressor enhancer of Lin12 1-like) in mammals and Hrd3 (HMG-CoA reductase degradation protein 3) in yeast [75]. Yos9/OS9 works together with Hrd3/Sel1L to bring a terminally misfolded glycoprotein to the ER membrane-anchored ERAD machinery that builds around a multi-transmembrane ubiquitin E3 ligase. In yeast, Hrd1 (HMG-CoA reductase degradation protein 1) and Doa10 (degradation of alpha2) are two major ERAD E3 ligases. It is generally believed that misfolded proteins with folding lesions in their luminal domains (ERAD-L) or membrane-spanning domains (ERAD-M) are degraded by Hrd1, and those defective in the cytoplasmic domains (ERAD-C) are mediated by Doa10 [76]. In fact, the ERAD systems that dispose a wide variety of substrates are more complicated in mammals that have many different E3 ubiquitin ligases implicated in ERAD [77]. It is worth mentioning that the yeast ERAD E3 ubiquitin ligases not only catalyze the polyubiquitin conjugation on their clients but also serve as retrotranslocons to extract ERAD substrates into the cytosol [78,79,80]. The ERAD retrotranslocons could also include several other transmembrane proteins, such as Der1 (degradation in the endoplasmic reticulum protein 1) [81] and its homolog DFM1 (DER1-like family member protein 1) [82]. Both the Hrd1- and Doa10-based ERAD machinery contain other conserved components, such as the AAA+-type ATPase, Cdc48 (cell division cycle protein 48, p97 in mammals) and its cofactors Npl4 (nuclear protein localization protein 4) and Ufd1 (ubiquitin fusion degradation protein 1) for escorting the extracted ERAD substrates to the proteasome, Cue1 (coupling of ubiquitin conjugation to ER degradation protein 1) for recruiting E2 and several ubiquitin-conjugating E2 enzymes [83] (Figure 1). Notably, while the α-1,6-mannose-exposed N-glycan structure serves as the ERAD signal for degrading misfolded glycoproteins, the recognition and targeting of nonglycosylated misfolded proteins for degradation remain unclear.

Previous studies have revealed that ERAD is important for plant growth and development [84,85], but the exact mechanisms were not well-understood until some endogenous ERAD substrates were discovered in plants. Most of the published plant ERAD studies were focused on mutant plant proteins known to be important for plant growth and stress tolerance. For example, several mutant variants of the barley mildew resistance Locus O (MLO) carrying single amino acid substitution in the cytosolic loops were discovered as plant ERAD clients degraded through the Hrd1-containing ERAD pathway involving the ubiquitin-conjugating enzyme UBC32 [45,86]. In Arabidopsis, two mutant variants of the brassinosteroid receptor BRI1 (BRASSINOSTEROID-INSENSITIVE 1), bri1-5 and bri1-9, are also degraded by the Hrd1 ERAD complex, producing the BR-insensitive dwarfism phenotype [87]. Forward and reverse genetic studies using the corresponding Arabidopsis *bri1-5* and *bri1-9* have discovered not only conserved but also plant-specific components of the Arabidopsis Hrd1-containing ERAD machinery (Table 1). The conserved components include EBS5 for EMS-mutagenized bri1 suppressor 5 (also known as AtHRD3A or AtSel1L for its sequence homology to Hrd3/Sel1L) [39,40], EBS6 (also known as AtOS9 for being the Arabidopsis homolog of Yos9/OS9) [41,42], Hrd1A and Hrd1B (two Arabidopsis homologs of the yeast Hrd1) [39], and ubiquitin conjugase UBC32 [45]. The plant-specific components include EBS7, an ER membrane protein that is highly conserved in land plants, and two highly homologous proteins known as PAWH1/2 for protein associated with Hrd1. EBS7 is predicted to contain three transmembrane segments, while PAWHs have an AIM24 (altered inheritance of mitochondria protein 24) domain that is thought to be conserved in land plants and plays important roles in ER stress tolerance and the UPR and a C-terminal membrane anchor [88]. Interestingly, loss-of-function mutations in EBS7 cause rapid degradation of the two PAWHs and Hrd1. The simultaneous elimination of PAWH1 and PAWH2 also leads to rapid disappearance of EBS7 and Hrd1, suggesting important regulatory functions of the two plant-specific components of the Hrd1 ERAD complex [43]. Further studies are needed to fully understand how EBS7 and PAWHs coordinately regulate the protein stability and E3 ligase activity of the Hrd1. Mass spectrometry-based proteomic approaches coupled with reverse genetics of Arabidopsis could identify additional components, regulators, and, more importantly, endogenous substrates of the plant Hrd1-containing ERAD system.

Compared to the HRD1 complex, little is known about the roles of other E3 ligases in the plant ERAD system. The Arabidopsis genome encodes two homologs of DOA10 (AtDOA10A and AtDOA10B). It has been reported that AtDOA10A (SUD1 for SUPPRESSOR OF DRY2 DEFECTS1 or CER9 for ECERIFERUM9) is involved in the cuticle lipid biosynthesis, plant drought response, and ABA metabolism [48,89], but the precise mechanisms and corresponding substrates remain unknown. A recent study demonstrated that degradation of a GFP fusion protein with the Arabidopsis SQUALENE EPOXIDASE (AtSQE1), a rate-limiting sterol synthetic enzyme that converts squalene into 2,3-epoxysqualene, is mediated by AtDOA10A [49]. However, AtSQE1 is a correctly folded ER transmembrane protein and little is known how such a correctly folded ERAD substrate is recognized by the ERAD system. It also remains to be investigated whether or not AtDOA10A-facilitated AtSQE1 degradation requires additional components of the AtDOA10A system. Unlike AtDOA10A, AtDOA10B could be induced by ER stress and interacted with UBC32 when coexpressed in tobacco leaves. Interestingly, DOA10B only exists in the Brassicaceae species and was unable to complement the yeast *doa10* mutant, suggesting that AtDOA10B might have a more distinctive function than AtDOA10A in the ER [49]. In contrast to yeast that has two ER membrane-anchored E3 ligases, plants have additional ER membrane-anchored/associated E3 ligases implicated in ERAD, such as RMA1 (RING membrane-anchored 1) and the Arabidopsis cytosolic E3 ligase EMR (ERAD-mediating RING finger protein). The Rma1H1 (RING membrane-anchor 1 homolog 1) from hot pepper (*Capsicum annuum*) and three homologs of RMA1 (AtRMA1, AtRMA2 and AtRMA3) from Arabidopsis are involved in regulating the trafficking and protein abundance of aquaporin PIP2;1 (plasma membrane intrinsic protein 2;1) [50,51]. EMR that is induced by ER stress colocalizes with the ER membrane-anchored UBC32 and exhibits the ubiquitin ligase activity in vitro [52]. Although EMR knockdown partially rescued the *bri1-5* dwarfism phenotype, it remains to be investigated whether EMR is involved in the ERAD of bri1-5 and other endogenous ERAD substrates.

Maintaining protein homeostasis in a changing environment is vital for cell function and organismal viability. In recent years, the physiological functions of ERAD have been extensively studied in mammals and proven to be closely related to health and disease [90]. In plants, ERAD is thought to play an essential role in plant adaptation to biotic and abiotic stress. ERAD components, such as HRD1A/1B, EBS6/AtOS9, EBS7, and PAWH1/2, are involved in the degradation of misfolded EFR (elongation factor thermo-unstable/EF-Tu receptor), a plasma membrane-localized immune receptor that recognizes and binds the bacterial translation elongation factor EF-Tu [42,43,44]. A recent study also revealed that OsUBC45, a rice homolog of the Arabidopsis UBC32, functions as an ERAD component that regulates rice resistance against blast disease and bacterial blight [46]. Salt treatment leads to the accumulation of ubiquitinated proteins and induces the ER stress response [40]. Arabidopsis mutants of the Hrd1 ERAD complex exhibit activated UPR and altered tolerance to salt stress [39,42,43,44,45]. Furthermore, UBC32 was also implicated in drought and oxidative stress responses [91], and a *hrd1a hrd1b* double mutant exhibits reduced sensitivity to heat stress [92]. Of note, some ERAD components, such as AtOS9 and UBC32, were themselves degraded via a self-regulatory mechanism known as “the ER turning” to influence plant growth and stress adaptation [42,45,47,93].

## 4. Removal of ER Portions through ER-Phagy

As the largest intracellular organelle, the ER undergoes dynamic remodeling during the cell cycle to maintain its structural integrity and metabolic functions. The aggregates of misfolded proteins and/or excess/damaged ER segments, which cannot be degraded by the ERAD pathway, are eventually removed through ER-phagy or reticulophagy, a selective autophagy by which parts of the ER network are removed through lysosomal degradation [94]. Depending on how the substrates are delivered to the lysosomes/vacuoles, ER-phagy can be classified into two categories: macro-ER-phagy and micro-ER-phagy. In macro-ER-phagy, a precursor cisterna called phagophore elongates, expands, and finally closes to form a sealed double membrane structure, known as the autophagosome. Autophagosomes then bind and deliver their ER cargos to lysosomes/vacuoles for degradation. In contrast, micro-ER-phagy involves the direct piecemeal engulfment of abnormal ER fragments by endosomal and/or lysosomal/vacuolar invagination [10,95].

The ER-phagy connects the excessive/damaged ER fragments with autophagosomes through the ER-phagy receptors that interact with the ubiquitin-like ATG8 (autophagy-related protein 8), permitting continued incorporation of damaged ER segments into the ATG8-decorated phagophores. To date, a number of membrane and soluble ER-phagy receptors have been identified in *Saccharomyces cerevisiae* and mammalian cells [95,96]. Their differential distribution throughout the ER sheets, tubes, and perinuclear ER facilitates targeting various regions of the ER network to the autophagosome. In plants, only a few ER-phagy receptors have been discovered, such as ATI1 (ATG8-Interacting protein 1)/ATI2 (ATG8-Interacting protein 2), Sec62 (translocation protein Sec62), Rtn1 (Reticulon-1)/Rtn2 (Reticulon-2), C53, and RHD3 (Root Hair Defective 3) proteins [97] (Figure 1). ATI1 and ATI2 are two plant-specific ER-phagy receptors that interact with ATG8 [54]. They have a single transmembrane domain and long N-terminal intrinsically disordered regions (IDRs) containing functional AIMs. It has been reported that ATI1 and ATI2 are located in the ER membrane. However, upon being exposed to darkness (carbon starvation), ATI1 and ATI2 associate with starvation-induced spherical compartments that are subsequently delivered to the vacuole, suggesting that both ATI1 and ATI2 are involved in the selective degradation of certain proteins [54]. AGO1 (Argonaute1) is the key component of the RNA silencing pathway and plays an important role in host innate antiviral immunity [98], but plant viruses have evolved suppressors of RNA silencing to hijack the host AGO1 for their transmission [99]. In Arabidopsis, AGO1 interacts with ATI1 and ATI2 on the ER and is targeted to the vacuole by the induction of the polerovirus F-box P0 protein [55,100]. In addition, MSBP1 (Membrane Steroid Binding Protein 1) has also been identified as an ER-phagy cargo. MSBP1 is localized in the ER and interacts with both ATI1 and ATI2. Following carbon starvation and the application of concanamycin A, a V-ATPase inhibitor that stabilizes autophagic bodies, MSBP1 colocalizes with ATI1 and travels through the ER network to reach the vacuole for autophagic degradation [56]. It is worth noting that the ATI1-mediated ER-phagy pathway is not induced by ER stress [56], which appears to be a distinct mechanism from other plant ER-phagy pathways.

As discussed above, the ER stress that triggers the UPR stimulates the production of many protein chaperones and folding catalysts, which is often accompanied with ER expansion to increase the protein folding capacity. Upon relief of the ER stress, eukaryotic cells need to reduce not only the abundance of those UPR-induced proteins but also the size of the ER network. In mammals, the translocon component Sec62 plays a crucial role in the recovER-phagy, a specific ER-phagy mechanism that re-establishes the ER homeostasis [101]. AtSec62, the Arabidopsis homolog of the mammalian Sec62, contains three transmembrane domains (TMDs) and a C-terminus luminal domain [57]. Loss-of-function mutations in AtSec62 lead to ER stress, impair vegetative growth, cause defective pollen development, and reduce fertility. Importantly, AtSec62 interacts with the Arabidopsis ATG8e through its AIMs upon ER stress induction [57]. Moreover, the overexpression of *AtSec62* in Arabidopsis enhances recovery from ER stress, a function similar to the mammalian Sec62 [57,101]. Thus, AtSec62 most likely functions as a plant ER-phagy receptor regulating the ER protein homeostasis and plays an important role in plant tolerance to environmental stresses.

It was known that maintaining the ER homeostasis requires dynamic changes in the ER structure consisting of interconnected branching tubules and flatten sacs/sheets throughout the entire cytoplasm [102]. Reticulons (Rtns) are a highly conserved eukaryotic protein family that mainly promotes the ER curvature [103]. Maize Rtn1 and Rtn2 are two ER-localized reticulon proteins that promote ER homeostasis in the aleurone cells of the corn endosperm. Rtn1/Rtn2 interact with the maize ATG8a, and their interactions were known to be enhanced upon treatment with the ER stress-inducing chemicals [58]. Importantly, the aleurone vacuoles of the maize *rtn1* and *rtn2* mutants accumulate many cytoplasmic fragments, suggesting that maize Rtn1 and Rtn2 could function as potential receptors for autophagy-mediated ER turnover [58]. In Arabidopsis, RTNLB3 (RTN-like B protein 3) and RTNLB13 were reported to physically interact with RHD3, a well-studied ER membrane-anchored atlastin-related GTPase important for the root hair development on the ER tubules that maintain the ER shape [104,105]. A recent study suggested that RHD3 might function as an ER-phagy receptor for selective ER protein degradation under ER stress based on its physical interaction with an Arabidopsis ATG8 and the reduced ER stress sensitivity and defective ER-phagy of the *rhd3* mutant [59].

Arabidopsis C53 is a highly conserved soluble ER-phagy receptor discovered through an ATG8-based proteomic study [60]. Intriguingly, C53 localizes to the ER by forming a ternary receptor complex with the two key components of the ufmylation system known to be important for ER-phagy: UFL1 (ufmylation E3 ligase) and its substrate-recruitment adapter DDRGK1 (DDRGK domain-containing protein 1) [106]. Under normal conditions, C53 is kept in its inactive form by binding with another ubiquitin-like protein UFM1 (ufmylation modifier 1). However, certain ER stress-induced ribosome stalling transfers UFM1 to the translocon-associated ribosome via the UFL1-catalyzed ufmylation, thus freeing C53 for its binding to the phagophore-decorating ATG8 and activating the highly selective C53-mediated ER-phagy. Thus, the competitive binding of C53 with UFM1 and ATG8 plays a crucial role in coordinating the ribosome-associated protein quality control and selective autophagy [60]. Further investigation is necessary to fully understand the biochemical mechanism that links the UFL1-mediated ufmylation of ribosomal proteins with the C53-mediated ER-phagy.

## 5. Integrating ER Protein Homeostasis Strategies

The UPR and ERAD are two distinct but functionally connected cellular pathways that regulate ER homeostasis in response to cellular and environmental stress. Misfolded proteins accumulated in the ER are sensed via the UPR sensors, which activate several interdependent signaling cascades that reduce the influx of proteins into the ER and boost protein folding/refolding capacity [107]. Most genes encoding components and regulators of the ERAD machinery are also induced by the UPR [108,109], thus promoting the ERAD and ER-phagy activity to dispose of misfolded proteins, improperly assembled protein complexes, large protein aggregates, and/or damaged ER fragments. Mutants with defective ERAD components often exhibit constitutive UPR activation and are hypersensitive to ER stress due to the over-accumulation of misfolded proteins in the ER [43,44,110]. The UPR can also be activated by various environmental stresses that interfere with protein folding or reduce ERAD efficiency or certain physiological conditions that demand much higher folding capacity. A short-term UPR is beneficial for cell survival by restoring ER homeostasis, but sustained overactivation of the UPR might trigger cellular dysfunction or even cell death [111]. In mammals, IRE1α and ATF6 were found to be targeted for ERAD via the SEL1L-HRD1 protein complex, providing an effective autoregulatory strategy to restrain UPR hyperactivation [112,113], yet it remains unclear whether the protein stability of IRE1 homologs in yeast and plants is also regulated by the ERAD system. Recent studies have revealed that plant IRE1 plays an important role in coordinating the UPR and ER-phagy. In Arabidopsis, ER-phagy can be induced by ER stress-inducing chemicals, causing portions of the ER to be targeted to the vacuole [114]. Such a process likely involves AtIRE1B, because the loss-of-function *ire1b* mutation contained fewer ER stress-induced autophagosomes, compared to its wild-type control [114]. A recent study suggested that the RIDD but not the kinase activity of AtIRE1B is necessary for ER stress-induced autophagy via the degradation of the mRNAs of yet to be defined ER-phagy inhibitors [115]. In addition, many ER-phagy receptors are induced by UPR [101,116], suggesting that ER-phagy not only accelerates the turnover of the ER under stress conditions but also helps to restore the ER size and functions during the ER stress recovery period.

ERAD and ER-phagy are the two major intracellular proteolytic pathways that selectively degrade target proteins to maintain ER homeostasis. It has been suggested that ERAD mainly degrades detergent-soluble misfolded proteins in a substrate-specific manner, but ER-phagy is responsible for the degradation of detergent-insoluble protein aggregates and damaged/excess ER fragments [117]. How cells coordinate the two different degradation pathways remains a major question. The Z variant of human α-1 proteinase inhibitor (A1PiZ) is a misfolded protein that can be recognized and degraded by the ERAD system in yeast and humans [118,119]. However, excessive accumulation of A1PiZ, which aggregates in the ER, is targeted to the vacuole via an autophagy pathway [118], suggesting that cells could resort to distinct strategies to degrade misfolded proteins depending on the abundance of substrates. Similarly, it was found that the deficiency of SEL1L-HRD1 ERAD in β cells resulted in the accumulation of proinsulin in the ER as high molecular weight conformers, which activate the ER-phagy pathway to eliminate these misfolded proinsulin proteins and their aggregates. However, when both SEL1L and autophagy are absent in β cells, mice develop diabetes shortly after weaning and die prematurely [120], suggesting that the induction of ER-phagy in the absence of ERAD might be a beneficial adaptation for cells to cope with the abnormal accumulation of misfolded proteins in the ER. Furthermore, it was revealed that SEL1L-HRD1 ERAD may have an impact on ER-phagy activity by limiting the availability of its substrate (ER fragments). It is only when SEL1L-HRD1 ERAD is compromised that the ER will be fragmented and then eliminated through ER-phagy [121]. CDC48 plays a vital role in many cellular processes that are essential for cell viability [53,122]. In ERAD, CDC48 is required for extracting ubiquitinated substrates from ER membranes for proteasomal degradation [83]. Interestingly, CDC48 is shown to be involved in the autophagy pathway, with CDC48 and its cofactor Shp1/Ubx1 being identified as essential components for the biogenesis of autophagosomes [123]. A recent study has found that non-functional CDC48 complexes can be eliminated through UIM-directed autophagy, which relies on the ubiquitin-interacting motif (UIM) sequences to bind to ATG8 [124,125]. In Arabidopsis, plant UBX domain-containing (PUX) protein PUX7, 8, 9, and 13 bind to ATG8 with their UIM motifs, thus allowing for the autophagic degradation of the inactive CDC48 [125]. Given the essential role of CDC48 in ERAD, further study is necessary to ascertain whether the ERAD pathway is regulated through autophagy via the CDC48 protein.

## 6. Concluding Remarks

Eukaryotes have evolved diverse ER quality control systems to maintain ER homeostasis. In recent years, despite the rapid progress made in understanding the processes and functions of the UPR, ERAD, and ER-phagy in yeast and mammalian cells, there are still many unanswered questions about these processes and functions in plants. Firstly, plants have developed conserved but different mechanisms to confront ER stress in response to changing environments. Further studies are needed to understand the molecular mechanisms of plant-specific proteins in the UPR pathway and to explore both their roles in controlling the expression of multiple UPR genes and their impact on cellular functions. In particular, the integration of the UPR with multiple plant physiological signals needs to be investigated. Secondly, the UPR and ERAD are interconnected processes that work together to maintain proteome integrity in the ER, and the crosstalk between the UPR and ERAD involves multiple regulatory mechanisms that require further investigation. In addition, it remains unclear when a misfolded protein terminates its futile repair process and enters into the ERAD pathway. Thirdly, plants have specific ERAD components that play essential roles in maintaining the stability of the Hrd1 complex, and elucidating the biological function and mechanistic details of these plant-specific components will help us to better understand the plant ERAD system. Meanwhile, recent advances in cryo-EM and proximity labeling proteomics offer the potential to explore the mechanisms underlying substrate recognition, retrotranslocation, and ubiquitin processing. In addition to the degradation of misfolded proteins, the ERAD system is also responsible for the quantity control of folding-competent proteins. Therefore, the plant endogenous substrates that are mediated by ERAD should be identified in future studies. Finally, the specific interplay and regulatory mechanisms between ERAD and ER-phagy in plants are not yet fully understood. Further research is needed to elucidate how ERAD may influence or be influenced by ER-phagy and to determine the molecular components and signaling pathways involved in this crosstalk.

## Figures and Tables

**Table 1:** Plant components involved in maintaining ER protein homeostasis.

Pathways	Name	Type	Targets	Species	References
UPR	AtIRE1A, AtIRE1B, AtIRE1C	Sensor	--	*A. thaliana*	[20,25,26]
	AtbZIP60	Transcription factor	--	*A. thaliana*	[23]
	AtbZIP17	Transcription factor	--	*A. thaliana*	[31]
	AtbZIP28	Transcription factor	--	*A. thaliana*	[30]
	S1P, S2P	Proteases	--	*A. thaliana*	[30]
	NAC	Transcription factor	--	*A. thaliana*	[34,35,36]
	DUF538	--	--	*A. thaliana*	[37,38]
ERAD	EBS5/AtHRD3A	Adaptor	MLO1; bri1-5/1-9	*A. thaliana*	[39,40]
	EBS6/AtOS9	Adaptor	EFR *; bri1-5/1-9	*A. thaliana*	[41,42]
	EBS7	--	EFR *; bri1-5/1-9	*A. thaliana*	[43]
	PAWH1, PAWH2	--	EFR *; bri1-5/1-9	*A. thaliana*	[44]
	UBC32	E2	MLO12; bri1-5/1-9	*A. thaliana*	[45]
	OsUBC45	E2	--	*O. sativa*	[46]
	HRD1A	E3 ligase	bri1-5/1-9; UBC32	*A. thaliana*	[39,47]
	AtDOA10A/CER9/SUD1	E3 ligase	HMGR; SQE1	*A. thaliana*	[48,49]
	AtRMA1, AtRMA2, AtRMA3	E3 ligase	PIP2;1	*A. thaliana*	[50,51]
	CaRma1H1	E3 ligase	PIP2;1	*C. annuum*	[50]
	EMR	E3 ligase	MLO12; bri1-5	*A. thaliana*	[52]
	CDC48A	AAA ATPase	--	*A. thaliana*	[53]
ER-phagy	ATI1, ATI2	Receptor	AGO1; MSBP1	*A. thaliana*	[54,55,56]
	AtSec62	Receptor	--	*A. thaliana*	[57]
	Rtn1, Rtn2	Receptor	--	*Z. mays*	[58]
	RHD3	Receptor	--	*A. thaliana*	[59]
	C53	Receptor	--	*A. thaliana*	[60]

*: misfolded EFR; and --: unknown.

## Data Availability

No new data were created or analyzed in this study. Data sharing is not applicable to this article.
